# Delay in the Diagnosis and Treatment of Oral Cancer

**Published:** 2013-09

**Authors:** A Jafari, SH Najafi, F Moradi, MJ Kharazifard, MR Khami

**Affiliations:** a Dept. of Dental Research Center and Community Oral Health, School of Dentistry, Tehran University of Medical Sciences, Tehran, Iran.; b Dept. of Oral Medicine, International Campus, School of Dentistry, Tehran University of Medical Sciences, Tehran, Iran.; c Dept. of Dental Research Center and Oral Medicine, School of Dentistry, Tehran University of Medical Sciences, Tehran, Iran; d Postgraduate Student, School of Dentistry, Yazd University of Medical Sciences, Yazd, Iran.; e Statistical Consultant, School of Dentistry, Tehran University of Medical Sciences. Tehran, Iran.

**Keywords:** Oropharyngeal Cancer, Patients Delay, Professional Delay

## Abstract

**Statement of Problem:** Oral and pharyngeal cancer is one of the most mortal cancers; however, its quick diagnosis and referral is a crucial factor in enhancing the survival rate of the patients.

**Purpose:** The aim of this study was to inspect the referral conditions and the reasons for the delay in curing the patients referred to the educational hospitals in Tehran.

**Materials and Method:** In this retrospective -descriptive study, two hundred and fifty six files related to the oral and pharyngeal cancer were inspected. The documents were obtained from 5 educational hospitals specialized in the field of cancers. Eventually data related to the time difference between the first time of attending to lesion and diagnosing the cancer as patient’s delay and until the curing as professional’s delay were recorded.

**Results:** The majority of cancers were squamous cell carcinoma (SCC). The patient’s delay was recorded in 110 files among the whole files. The mean of the time between the patients’ first notice of the problem and the time visiting a primary care clinician was 270 days (range, 0-2520 days). The mean of the time from when the patient visited a primary- care clinician to the starting time of definitive treatment was 90 days (range, 0-270 days).

**Conclusion:** In this study, like other studies, SCC was the most common occurring cancer. Delays related to the patients were more than those related to the professionals. And at last, accuracy in recording the files and training the patients were recognized to be the most imperative factors to continue the treatment successfully.

## Introduction

Cancers are generally categorized as one of the most major problems of human beings; however, oral cancer, especially in some specific regions such as South and Central Asia, is a recognized problem [1]. Oral cancer has one of the lowest five-year survival rate among the major types of cancers, including breast, skin, testis, prostate, uterus, and urinary bladder cancers [2] with survival rates of 50% or less [3]. Early diagnosis is crucial for improving the survival rate. If the detected lesions are small localized and treated efficiently; survival rates of 70 to 90% can be achieved [4]. The five-year survival rate for persons with localized lesions is four times greater than with the lesions with distant metastases [3]. Although oral cancer occurs in a part of the body that is readily accessible for early detection, most lesions are not diagnosed until they have reached advanced stages. For example SCC in oropharynx has the same symptoms as pharyngitis and viral tonsillitis, and it may be confused with these diseases if the involved patients have been visited by a general practitioner and consequently this may be one of the main reasons of diagnosis and treatment delay. 

Cancer is the third major cause of death in Iran, and more than thirty thousand people lose their lives annually [4]. A study done in Tehran rated the 5- year- survival rate to be 30%, which is much less than its worldwide average rate [5]. The delay time in diagnosing and treating the cancers in different studies is about 2 to 5 months [6]. The statistics related to the incidence of cancers illustrates more than 70000 new cases of cancer annually in Iran [4]. The aim of this study was to consider the delay in diagnosing and treating the oral and pharyngeal cancers during a 5- year period in specified cancer research centers of Tehran. 

## Material and Methods

This study was an observation descriptive-analytic study. Five educational hospitals and specialized cancer curing centers were chosen. The centers were chosen based on their specialty on treating oral and pharyngeal cancers. In this study all existing archived files related to oral and pharyngeal cancer were examined in a 5- year period between 2001 and 2005 to explore the demographic features of the patients suffering from malignant oral and pharyngeal tumors and the way they were diagnosed with histopathologically confirmed squamous cell oral or pharyngeal cancer (ICDO141, 143-145, 148-149) and treated. Therefore, we evaluated factors like gender, age, marital status, ethnic background, occupation or activity, patient delay and professional delay. Among all the existing files in the five centers, 256 files having expected data were selected to be analyzed at the final stage and repetitive ones were analyzed after being collected from different centers. Two methods were used in order to collect the information: filling out the pre-prepared forms based on the archived files and completing the partial files and adding data by calling the patients’ families. ICDO-coding system [7] was used to determine the kind of malignant lesions, and Diz Dios system was used to determine patients’ treatment delay that was divided into two groups; patient delay and practitioner delay [8].

Patient delay is defined as the length of time, in days, from the time at which the patient first became aware of symptoms (subsequently shown to be oral cancer) to his or her first visit to a primary care clinician (physician or dentist). This time often was approximate because patients tended to estimate time in months, but sometimes they could identify the week or day when they first became aware of the cancer [9].

 Professional delay, broadly defined, is the time between the patient entering professional care and the start of definitive treatment. There are time thresholds for each step in this process, above which the professional is at fault for delay [10]. The patients have mostly estimated the delay by months, unless the delay was less than a month and they would have daily or weekly estimations. The gap between examining the patient by a specialist and diagnosing the cancer, and the time when the patient is being treated was classified as the delay for which the professional is responsible [11]. The forms, prepared to record the data, were analyzed by SPSS software after being filled out. Frequencies of each variable and some relation between them were analyzed.

Effects of the variables on the patient delay and professional delay were analyzed using multiple linear regressions by stepwise method. P value less than o.o5 were considered as significant. 

## Results

Among the 256 files explored in this study, 62.1% was related to males; and 62% of the patients were 50 years old and more. Gender was a significant factor in diagnostic delay so that delay for men was significantly greater than for women (*p*= 0.024).

There was no linear relation between increasing age and the time between observations and referrals. But when we divided the cases into 3 groups: less than 35 years, between 36 and 65, and more than 66 years old, the two groups; less than 35, and more than 66 years old, had less delay in time between observation and referral than middle group (*p*< 0.05).

By dividing cases to two categories, active and retired, delay in refer to medicine was significantly more in active group (*p*= 0.032). The most prevalent cancer among oral and pharyngeal cancers was SCC (70.3%) (table 1). In the field of diagnosing the cancer based on the stages, the study proved that 28% of the cancers were diagnosed at the first stage, and 22.6% of them were diagnosed in the second stage. While majority (34.4%) of the diagnoses were at fourth stage (roughly 34.4%). However, regarding the SCC; 53.3% of the cancers were diagnosed at the third and higher stages. The percentage of men whose cancers were diagnosed at the fourth stage (39%) was significantly more than women (31%). There was no significant difference between lesion stage and delay in diagnosis (*p*= 0.498). Inspecting the delays related to the patients, from the time when they observed the signs or symptoms of the illness until diagnosed as having the cancer, illustrated an average delay of 8.2 months. While, the average delay related to the specialist, from the time when the first signs were diagnosed or the laboratory test confirmed the cancer till treating the cancer, was 3 weeks (Table 2). 

**Table 1:** Frequency of oro-pharyngeal cancers and theirs age

**Cancer**	**Number**	**Min. age**	**Max. age**	**Mean age**
Squamus Cell Carcinoma	178	17	90	62.85
Basal Cell Carcinoma (Lip)	3	22	53	37.33
Mucoepidermoid Carcinoma	14	3	67	36.92
Adeno Carcinoma	11	29	90	54.90
Lymphoepithelial Carcinoma	1	49	49	49
Lymphoma	11	2	90	47.72
Osteo Sarcoma	2	50	53	51.50
Melanoma	9	24	76	51.33
Soft Tissue Sarcoma	4	18	65	36.75
Others or Unknowns	21	10	73	46.85

**Table 2:** Frequencies of patient’s delay and professional’s delay in month

	**No.**	**Min**	**Max**	**Mean**	**S D**
Patient’s delay	211	0	24	8.2	5.2
Professional’s delay	185	0	3	0.7	1.1

The delay related to the patients suffering from SCC was the same as the average delay period; however, the delay related to the practitioners was less than the average delay (3 weeks). Furthermore, lesions in tongue and pharyngeal areas were diagnosed later. The mean time was of 12 and 13 months. 

## Discussion

The average time period of delay varies in different populations and studies. It has been estimated that about 50% of patients with oral cancer make a first visit to a healthcare professional within 1-2 months of becoming aware of symptoms, while about 20-30% of patients delay seeking help for more than 3 months [12-13]. The mean time of delay in our study was 8.2 months. This study showed that the patient with higher stage cancer had more delay; it was the same as the other study [14]. The study showed that men had more delay than women. Paying more attention to the oral health and lesions existed in this area in women could be the reason why the delay related to men is more than the delay related to women. 

In this study, 74% of the malignant cases is seen in the patients with 40-65 years old. The worldwide studies prove that majority of the patients are more than 40 [15]. SCC is an age-related cancer, and it usually occurs after the age 50 [16]. The average age of the patients suffering from SCC in the worldwide studies is 62, which is similar to the Iranian patients’ average age [5]. Although incidence of oral cancer in Iran has been expressed as a low level in the world but the rate of mortality has been reported as a medium level [17]. This may be a cause of increasing the mortality compared with other countries [18]. Clinical factors include tumor size, tumor site, and tumor stage at the time of diagnosis, and lymph node metastasis. Most studies showed no relationship between patient delay and the stage of diagnosis [19]. Also, in our study, There was no significant difference between lesion stage and delay in diagnosis (*p*= 0.498). In the current study, patient delay periods were longer than practitioner delay. The reasons for this may vary.

One reason can be the financial problems of many patients who are at risk of developing oral cancer. The dental professions need to find a way to eliminate financial limitations that prevent patients from seeking care when symptoms first appear. Increased access to health care, including dental care, for uninsured patients would likely allow for more frequent detection of early-stage cancers.

Other factor that influence on the patient delay is education of the public. Health care providers must place greater emphasis on educating patients about the importance of visiting a clinician as soon as oral symptoms develop. They can visit a dentist or a physician and the clinician should schedule an early appointment for these patients. Public education efforts must also continue to encourage patients to avoid high-risk behaviors as tobacco or alcohol use.

One important concept, in increasing awareness of oral cancer is to institute a self-examination campaign similar to the monthly breast self-examination campaign. Patients without symptoms might visit a general dentist every six months and a physician every year. Any self-examination conducted between these intervals might result in early detection of the lesions and accelerate visits to the health care professionals before symptoms develop. A visual examination of the oral cavity in a mirror on a monthly basis would be fairly easy to perform and could result in the detection of some lesions. Patients would be instructed to lift up their tongues to view the floor of the mouth, move the tongue to the right and left of the mouth to evaluate the lateral surfaces and pull both cheeks laterally to examine the vestibules, gingivae and buccal mucosa. Self-examination has the potential to enable patients to detect asymptomatic cancers at early stages. Not only does self-examination increase the frequency with which the oral cavity is screened, but it is done at no burden to the health care system beyond patient education. 

In conclusion, in our study, the longest delay occurred between the patient’s first becoming aware of symptoms and visiting a primary care clinician. Health care professionals need to place more emphasis on early self-referrals, as well as accelerating the other parts of the referral process, particularly in referrals from a primary care clinician to a specialist. Furthermore, inspecting the posing obstacles in the path of recovering and increasing the patients’ lifespan must be an unforgettable task in the healthcare system. Government agencies, universities and dental clinics have made attempts to diagnose cancer early, via population screening and the use of various visual and chemical detection methods.

## References

[1] Mousavi SM, Donlou M, Haj Sadeghi N (2006). Guideline of records and report of cancer.

[2] Pisani P, Parkin DM, Bray F, Ferlay J (1999). Estimates of the worldwide mortality from 25 cancers in 1990. Int J Cancer.

[3] Clegg LX, MidthuneDN, Feuer, EJ Fay, MP Hankey BF, Ries LAG, Eisner MP, Kosary CL, Hankey BF, Clegg LX, Edwards BK (2000). Section XXIX: Cancer Incidence Rates Adjusted for Reporting Delay. SEER Cancer Statistics Review, 1973-1997.

[4] Mousavi SM, Ramazani R, Davanloo M (2008). National Cancer Registry Report 2005–2006.

[5] Sargeran K, Murtomaa H, Safavi SM, Vehkalahti M, Teronen O (2006). Malignant oral tumors in Iran: ten-year analysis on patient and tumor characteristics of 1042 patients in Tehran. J Craniofac Surg.

[6] Onizawa K, Nishihara K, Yamagata K, Yusa H, Yanagawa T, Yoshida H (2003). Factors associated with diagnostic delay of oral squamous cell carcinoma. Oral Oncol.

[7] Florian L, Wilhelm G (2006). Health Informatics, medical data management: a practical Guide. Springer London.

[8] Diz Dios P, Padrón González N, Seoane Lestón J, Tomás Carmona I, Limeres Posse J, Varela-Centelles P (2005). "Scheduling delay" in oral cancer diagnosis: a new protagonist. Oral Oncol.

[9] Onizawa K, Nishihara K, Yamagata K, Yusa H, Yanagawa T, Yoshida H (2003). Factors associated with diagnostic delay of oral squamous cell carcinoma. Oral Oncol.

[10] Hollows P, McAndrew PG, Perini MG (2000). Delays in the referral and treatment of oral squamous cell carcinoma. Br Dent J.

[11] Donnell A, Jin S, Zavras AI (2008). Delay in the diagnosis of oral cancer. J Stomatol Invest.

[12] Kerdpon D, Sriplung H (2001). Factors related to advanced stage oral squamous cell carcinoma in southern Thailand. Oral Oncol.

[13] Allison P, Franco E, Black M, Feine J (1998). The role of professional diagnostic delays in the prognosis of upper aerodigestive tract carcinoma. Oral Oncol.

[14] Pitiphat W, Diehl SR, Laskaris G, Cartsos V, Douglass CW, Zavras AI (2002). Factors associated with delay in the diagnosis of oral cancer. J Dent Res.

[15] Parkin DM, Bray F, Ferlay J, Pisani P (2005). Global cancer statistics, 2002. CA Cancer J Clin.

[16] Chen GS, Chen CH (1995). A statistical analysis of oral squa- mous cell carcinoma. Kaohsiung J Med Sci.

[17] Petersen PE (2009). Oral cancer prevention and control the approach of the World Health Organization. Oral Oncol.

[18] Donnell A, Jin S, Zavras AI (2008). Delay in the diagnosis of oral cancer. J Stomatol Invest.

[19] Kerdpon D, Sriplung H (2001). Factors related to advanced stage oral squamous cell carcinoma in southern Thailand. Oral Oncol.

